# The maximum clique enumeration problem: algorithms, applications, and implementations

**DOI:** 10.1186/1471-2105-13-S10-S5

**Published:** 2012-06-25

**Authors:** John D Eblen, Charles A Phillips, Gary L Rogers, Michael A Langston

**Affiliations:** 1 Center for Molecular Biophysics, Oak Ridge National Laboratory, Oak Ridge, TN 37831, USA; 2 Department of Electrical Engineering and Computer Science, University of Tennessee, Knoxville, TN 37996-3450, USA

## Abstract

**Background:**

The maximum clique enumeration (MCE) problem asks that we identify all maximum cliques in a finite, simple graph. MCE is closely related to two other well-known and widely-studied problems: the maximum clique optimization problem, which asks us to determine the size of a largest clique, and the maximal clique enumeration problem, which asks that we compile a listing of all maximal cliques. Naturally, these three problems are NP-hard, given that they subsume the classic version of the NP-complete clique decision problem. MCE can be solved in principle with standard enumeration methods due to Bron, Kerbosch, Kose and others. Unfortunately, these techniques are ill-suited to graphs encountered in our applications. We must solve MCE on instances deeply seeded in data mining and computational biology, where high-throughput data capture often creates graphs of extreme size and density. MCE can also be solved in principle using more modern algorithms based in part on vertex cover and the theory of fixed-parameter tractability (FPT). While FPT is an improvement, these algorithms too can fail to scale sufficiently well as the sizes and densities of our datasets grow.

**Results:**

An extensive testbed of benchmark graphs are created using publicly available transcriptomic datasets from the Gene Expression Omnibus (GEO). Empirical testing reveals crucial but latent features of such high-throughput biological data. In turn, it is shown that these features distinguish real data from random data intended to reproduce salient topological features. In particular, with real data there tends to be an unusually high degree of maximum clique overlap. Armed with this knowledge, novel decomposition strategies are tuned to the data and coupled with the best FPT MCE implementations.

**Conclusions:**

Several algorithmic improvements to MCE are made which progressively decrease the run time on graphs in the testbed. Frequently the final runtime improvement is several orders of magnitude. As a result, instances which were once prohibitively time-consuming to solve are brought into the domain of realistic feasibility.

## Background

A clique is a fully-connected subgraph in a finite, simple graph. The problem of determining whether or not a graph has a clique of a given size, called simply CLIQUE, is one of the best known and most widely studied combinatorial problems. Although classically formulated as an NP-complete decision problem [1], where one is merely asked to determine the existence of a certain size clique, the search and optimization formulations are probably most often encountered in practice, where one is asked to find a clique of given size and largest size respectively. In computational biology, one needs to look no farther than PubMed to gauge clique's utility in a variety of applications. A notable example is the search for putative molecular response networks in high-throughput biological data. Popular clique-centric tools include clique community algorithms for clustering [2] and paraclique-based methods for QTL analysis and noise abatement [3,4].

A clique is *maximal *if it cannot be augmented by adding additional vertices. A clique is *maximum *if it is of largest size. A maximum clique is particularly useful in our work on graphs derived from biological datasets. It provides a dense core that can be extended to produce plausible biological networks [5]. Other biological applications include the thresholding of normalized microarray data [6,7], searching for common *cis*-regulatory elements [8], and solving the compatibility problem in phylogeny [9]. See [10] for a survey of additional applications of maximum clique.

Any algorithm that relies on maximum clique, however, has the potential for inconsistency. This is because graphs often have more than just one maximum clique. Idiosyncrasies between algorithms, or even among different implementations of the same algorithm, are apt to lead to an arbitrary choice of cliques. This motivates us to find an efficient mechanism to enumerate all maximum cliques in a graph. These can then be examined using a variety of relevant criteria, for example, by the average weight of correlations driven by strain or stimulus [11].

We therefore seek to solve the *Maximum Clique Enumeration (MCE) *problem. Unlike maximal clique enumeration, for which a substantial body of literature exists, very little seems to be known about MCE. The only exception we have found is a game-theoretic approach for locating a predetermined number of largest cliques [12].

While very little prior work seems to have been done on MCE, the problem of maximal clique enumeration has been studied extensively. Since any algorithm that enumerates all maximal cliques also enumerates all maximum cliques, it is reasonable to approach MCE by attempting first to adapt existing maximal clique enumeration algorithms. An implementation of an existing maximal clique enumeration algorithm also provides a useful runtime benchmark that should be improved upon by any new approach. Besides maximal clique enumeration algorithms, another potential strategy is to compute the maximum clique size and then test all possible combinations of vertices of that size for connectivity. While this approach may be reasonable for very small clique sizes, as the maximum clique size increases the runtime quickly becomes prohibitive, and we mention it only for completeness, and focus our efforts on modifying and extending existing algorithms for enumerating maximal cliques.

**Figure 1:**
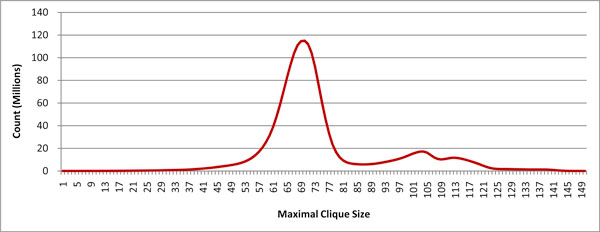
**Maximal Clique Profile**. The maximal clique profile of a graph created from the GDS3672 dataset using a threshold value of 0.81, the dataset's second highest threshold. MCE algorithms that are based on a breadth-first traversal of the search tree will retain at each step all maximal cliques of a given size. This can lead to titanic memory requirements. This graph, for example, contains more than 110 million maximal cliques of size 70. These sort of memory demands tend to render non-backtracking methods impractical.

Current maximal clique enumeration algorithms can be classified into two general types: iterative enumeration (breadth-first traversal of a search tree) and backtracking (depth-first traversal of a search tree). Iterative enumeration algorithms, such as the method suggested by Kose *et al *[13], enumerate all cliques of size *k *at each stage, test each one for maximality, then use the remaining cliques of size *k *to build cliques of size *k *+ 1. The process is typically initialized for *k *= 3 by enumerating all vertex subsets of size 3 and testing for connectivity. In practice, such an approach can have staggering memory requirements, because all cliques of a given size must be retained at each step. In [14], this approach is improved by using efficient bitwise operations to prune the number of cliques that must be saved. Nevertheless, storage needs can be excessive, since all maximal cliques of one size must still be made available before moving on to the next larger size. Figure 1 shows the number of maximal cliques of each size in one of the graphs near the median size in our testbed. This graphic illustrates the enormous lower bounds on memory that can be encountered with iterative enumeration algorithms.

Many variations of backtracking algorithms for maximal clique enumeration have been published in the literature. To the best of our knowledge, all can be traced back to the algorithms of Bron and Kerbosch first presented in [15]. Some subsequent modifications tweak the data structures used. Others change the order in which vertices are traversed. See [16] for a performance comparison between several variations of backtracking algorithms. As a basis for improvement, however, we implemented the original, highly efficient algorithm of [15]. We made this choice for three reasons. First, an enormous proportion of the time consumed by enumeration algorithms is spent in outputting the maximal cliques that are generated. This output time is a practical limitation on any such approach. Second, a graph can theoretically contain as many as 3^(*n*/3) ^maximal cliques [17]. It was shown in [18] that the algorithm in [15] achieves this bound in the worst case. No algorithm with a theoretically lower asymptotic runtime can thus exist. Third, and most importantly, the improvements we introduce do not depend on the particulars of any one backtracking algorithm; they can be used in conjunction with any and all of them.

## Results and discussion

Using the seminal maximal clique enumeration algorithm due to Bron and Kerbosch [15] as a benchmark, we designed, implemented, and extensively tested three algorithmic improvements, the last based on observations about the nature of graphs produced by transcriptomic data. Along with describing these improvements, we will describe our existing tool for finding a single maximum clique, based on the theory of fixed-parameter tractability (FPT) [19,20]. Such a tool is essential for all three improvements, since the first two rely on knowledge of the maximum clique size, and the last uses the maximum clique finding tool as a subroutine. All codes are written in C/C++ and compiled in Linux. For testing, we use 100 graphs derived from 25 different datasets which are publicly available on GEO. We concentrate on transcriptomic data, given its abundance, and eschew synthetic data, having learned long ago that effective algorithms for one have little bearing on the other. (The pathological matchings noted in [21] for vertex cover can be extended to clique, but likewise they too are of course hugely irrelevant to real data.) In an effort to improve performance, we scrutinize the structure of transcriptomic graphs and explore the notion of maximum clique covers and essential vertex sets. Indeed, we find that with the right preprocessing we are able to tailor algorithms to the sorts of data we routinely encounter, and that we can now solve instances previously considered unassailable.

### Algorithms

In the following sections, we describe each of the MCE algorithms we implemented and tested. The first is the algorithm of Bron and Kerbosch [15], which we call *Basic Backtracking *and use as a benchmark. Since all our subsequent improvements make use of an algorithm that finds a single maximum clique, we next describe our existing tool, called *Maximum Clique Finder (MCF)*, which does just that. We next modify the Basic Backtracking algorithm to take advantage of the fact that we only want to find the maximum cliques and can quickly compute the maximum clique size. We call this approach *Intelligent Backtracking*, since it actively returns early from branches that will not lead to a maximum clique. We then modify MCF itself to enumerate all maximum cliques, an approach we call *Parameterized Maximum Clique*, or *Parameterized MC*. In a sense this is another backtracking approach that goes even further to exploit the fact that we only want to find maximum cliques. Finally, based on observations about the properties of biological graphs, we introduce the concepts *maximum clique covers *and *essential vertex sets*, and apply them to significantly improve the runtime of backtracking algorithms.

#### Basic backtracking

The seminal maximal clique publication [15] describes two algorithms. A detailed presentation of the second, which is an improved version of the first, is provided. It is this second, more efficient, method that we implement and test. We shall refer to it here as Basic Backtracking. All maximal cliques are enumerated with a depth-first search tree traversal. The primary data structures employed are three global sets of vertices: COMPSUB, CANDIDATES and NOT. COMPSUB contains the vertices in the current clique, and is initially empty. CANDIDATES contains unexplored vertices that can extend the current clique, and initially contains all vertices in the graph. NOT contains explored vertices that cannot extend the current clique, and is initially empty. Each recursive call performs three steps:

• Select a vertex *v *in CANDIDATES and move it to COMPSUB.

• Remove all vertices not adjacent to *v *from both CANDIDATES and NOT. At this point, if both CANDIDATES and NOT are empty, then COMPSUB is a maximal clique. If so, output COMPSUB as a maximal cique and continue the next step. If not, then recursively call the previous step.

• Move *v *from COMPSUB to NOT.

Note that NOT is used to keep from generating duplicate maximal cliques. The search tree can be pruned by terminating a branch early if some vertex of NOT is connected to all vertices of CANDIDATES.

Vertices are selected in a way that causes this pruning to occur as soon as possible. We omit the details since they are not pertinent to our modifications of the algorithm.

The storage requirements of Basic Backtracking are relatively modest. No information about previous maximal cliques needs to be retained. In the improvements we will test, we focus on speed but also improve memory usage. Thus, such limitations are in no case prohibitive for any of our tested methods. Nevertheless, in some environments, memory utilization can be extreme. We refer the interested reader to [14].

Our Basic Backtracking implementation serves as an initial benchmark upon which we can now try to improve.

#### Finding a single maximum clique

We use the term Maximum Clique Finder (MCF) to denote the software we have implemented and refined for finding a single clique of largest size [22]. MCF employs a suite of preprocessing rules along with a branching strategy that mirrors the well-known FPT approach to vertex cover [19,23]. It first invokes a simple greedy heuristic to find a reasonably large clique rapidly. This clique is then used for preprocessing, since it puts a lower bound on the maximum clique size. The heuristic works by choosing the highest degree vertex, *v*, then choosing the highest degree neighbor of *v*. These two vertices form an initial clique *C*, which is then iteratively extended by choosing the highest degree vertex adjacent to all of *C*. On each iteration, any vertex not adjacent to all of *C *is removed. The process continues until no more vertices exist outside *C*. Since |*C*| is a lower bound on the maximum clique size, all vertices with degree less than |*C *- 1| can be permanently removed from the original graph. Next, all vertices with degree *n *- 1 are temporarily removed from the graph, but retained in a list since they must be part of any maximum clique. MCF exploits a novel form of color preprocessing [22], used previously in [24] to guide branching. This form of preprocessing attempts to reduce the graph as follows. Given a known lower bound *k *on the size of the maximum clique, for each vertex *v *we apply fast greedy coloring to *v *and its neighbors. If these vertices can be colored with fewer than *k *colors, then *v *cannot be part of a maximum clique and is removed from the graph. Once the graph is thus reduced, MCF uses standard recursive branching on vertices, where each branch assumes that the vertex either is or is not in the maximum clique.

#### Intelligent backtracking

**Figure 2:**
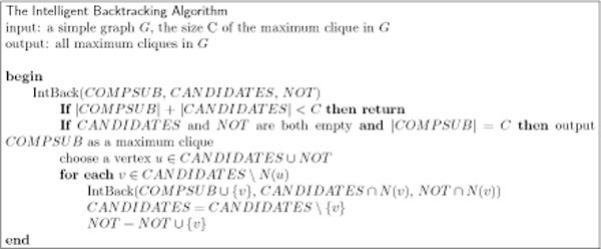
**Intelligent Backtracking**. A minor change to the Bron-Kerbosch algorithm uses the precomputed maximum clique size to trim the recursion tree. The input graph has typically been reduced using color preprocessing. %endfigure.

Given the relative effectiveness with which we can find a single maximum clique, it seems logical to consider whether knowledge of that clique's size can be helpful in enumerating all maximum cliques. As it turns out, knowledge of the maximum clique size *k *leads to a small, straightforward change in the Basic Backtracking algorithm. Specifically, at each node in the search tree we check if there are fewer than *k *vertices in the union of COMPSUB and CANDIDATES. If so, that branch cannot lead to a clique of size *k*, and so we return. See Figure 2. While the modification may seem minor, the resultant pruning of the search tree can lead to a substantial reduction in the search space. In addition to this minor change to branching, we apply color preprocessing as previously described to reduce the graph before submitting it to the improved backtracking algorithm. Color preprocessing combined with the minor branching change we call *Intelligent Backtracking*.

#### Paramaterized enumeration

Given that MCF employs a vertex branching strategy, we investigated whether it could be modified to enumerate not just one, but all maximum cliques. It turns out that MCF, also, lends itself to a straightforward modification that results in enumeration of all maximum cliques. The modification is simply to maintain a global list of all cliques of the largest size found thus far. Whenever a larger maximum clique is found, the list is flushed and refreshed to contain only the new maximum clique. When the search space has been exhausted, the list of maximum cliques is output.

We must take special care, however, to note that certain preprocessing rules used during interleaving are no longer valid. Consider, for example, the removal of a leaf vertex. The clique analogue is to find a vertex with degree *n *- 2 and remove its lone non-neighbor. This rule patently assumes that only a single maximum clique is desired, because it ignores any clique depending on the discarded vertex. Therefore this particular preprocessing rule must be omitted once branching has begun.

#### Maximum clique covers

If we view MCF as a black box subroutine that can be called repeatedly, it can be used in a simple greedy algorithm for computing a maximal set of disjoint maximum cliques. We merely compute a maximum clique, remove it from the graph, and iterate until the size of a maximum clique decreases. To explore the advantages of computing such a set, we introduce the following notion:

**Definition 1 ***A *maximum clique cover *of G = *(*V, E*) *is a set V' *⊆ *V with the property that each maximum clique of G contains some vertex in the cover*.

The union of all vertices contained in a maximal set of disjoint maximum cliques is of course a maximum clique cover (henceforth MCC), because all maximum cliques must overlap with such a set. This leads to a useful reduction algorithm. Any vertex not adjacent to at least one member of an MCC cannot be in a maximum clique, and can thus be removed.

In practice, we find that applying MCC before the earlier backtracking algorithms yields only marginal improvement. The concept of MCC does, however, lead to a much more powerful approach based on individual vertices. Since any improvement made by MCC is subsumed by the next approach, we do not test MCC by itself.

#### Essential vertex sets

Our investigation of the MCC algorithm revealed that it typically does not reduce the size of the graph more than the preprocessing rules already incorporated into MCF. For example, MCF already quickly finds a lower bound on the maximum clique size and removes any vertex with degree lower than this bound. Upon closer examination, however, we found that for 74 of 75 graphs that we initially tested for the conference version of this paper, only one clique was needed in an MCC. That is to say, one maximum clique covered all other maximum cliques. And in our current testbed of 100 graphs, in every case a single maximum clique suffices for an MCC. In fact this coincides closely with our experience, in which we typically see high overlap among large cliques in the transcriptomic graphs we encounter on a regular basis. Based on this observation, we shall now refine the concept of MCC. Rather than covering maximum cliques with cliques, we cover maximum cliques with individual vertices.

We define an *essential vertex *as one that is contained in every maximum clique. Of course it is possible for a given graph to have no such vertex, even when it contains many overlapping maximum cliques. But empirical testing of large transcriptomic graphs shows that an overwhelming number contain numerous essential vertices. And for purposes of reducing the graph, even one will suffice. An essential vertex has the potential to be extremely helpful, because it allows us to remove all its non-neighbors. We employ the following observation: for any graph *G, ω*(*G*) >*ω*(*G*/*v*) if and only if *v *covers all maximum cliques, where *ω*(*G*) is the maximum clique size of *G*.

**Figure 3:**
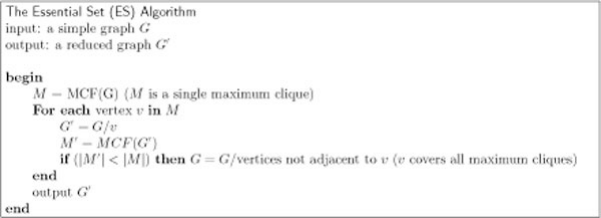
**The Essential Set (ES) Algorithm**. The ES algorithm finds all essential vertices in a graph and removes their non-neighbors.

We define an *essential set *to be the set of all essential vertices. The Essential Set (ES) algorithm, as described in Figure 3, finds all essential vertices in a graph. It then reduces the graph by removing, for each essential vertex, all non-neighbors of that vertex. The ES algorithm can be run in conjunction with any of the backtracking MCE algorithms, or indeed prior to any algorithm that does MCE by any method, since its output is a reduced graph that still contains all maximum cliques from the original graph. As our tests show, the runtime improvement offered by the ES algorithm can be dramatic.

### Implementation

We implemented all algorithms in either C or C++. The code was compiled using the GCC 4.4.3 compiler on the Ubuntu Linux version 10.04.2 operating system as well as the GCC 3.3.5 compiler under Debian Linux version 3.1. All timings were conducted in the latter Debian environment on dedicated nodes of a cluster to ensure no affect on timings from concurrent processes. Each node had a dual-core Intel Xeon processor running at 3.20 GHz and 4 GB of main memory.

### Testing

In the conference version of this paper, we used three different datasets at 25 thresholds each to derive a total of 75 graphs on which to test our algorithmic improvements. While these graphs certainly sufficed as an initial proof of concept, two concerns could be raised regarding them. First, one might argue that three datasets are not a sufficiently large sample size to provide a true sense of the overall nature of transcriptomic data or an algorithmic improvement's general effectiveness on such data, the large number of thresholds notwithstanding. And second, since the three datasets are proprietary and not publicly available, the results were not as readily reproducible as they might otherwise have been. Obtaining de-identified versions, while feasible, was an unnecessary obtacle to reproducibility.

We address such concerns here by creating a new suite of transcriptomic graphs on which to test our algorithmic improvements. The suite consists of graphs derived from 25 datasets obtained from the Gene Expression Omnibus (GEO) [25], a publicly accessible repository. For each dataset, graphs were created at four different thresholds, for a total of 100 graphs. The datasets were selected to provide a reasonably diverse sampling of experimental type, species, and mRNA microarray chip type. They cover 8 different species and a number of different experimental conditions such as time series, strain, dose, and patient. Since our graphs are derived from thresholding correlation values, we excluded from consideration any dataset with fewer than 12 conditions. Thresholding correlations calculated using so few conditions can produce unacceptably large rates of false positives and false negatives. The number of conditions range from a low of 12 to a high of 153. Nine of the datasets had not been log-transformed, in which case we performed log-transformation. Four of the datasets contained missing values; in these cases we used correlation p-values rather than correlations for the threshold. See Table 1 for a listing of the GEO datasets used for testing.

**Table 1:** GEO Datasets Used for Testing

DataSet	Title	Organism
GDS3505	Seedling roots response to auxin and ethylene availability	Arabidopsis thaliana
GDS3521	Retina response to hypoxia and subsequent reoxygenation: time course	Mus musculus
GDS3538	Age and diet effect on canine skeletal muscles	Canis lupus familiaris
GDS3561	Occupational benzene exposure: peripheral blood mononuclear cells (HumanRef-8)	Homo sapiens
GDS3579	Fer-1 null mutants	Caenorhabditis elegans
GDS3592	Ovarian normal surface epithelia and ovarian cancer epithelial cells	Homo sapiens
GDS3595	Macrophage response to H1N1 and H5N1 influenza viral infections	Homo sapiens
GDS3603	Renal cancer response to rapamycin analog CCI-779 treatment:	Homo sapiens
GDS3605	Spared nerve injury model of peripheral neuropathic pain: dorsal horn of spinal cord	Rattus norvegicus
GDS3610	Nasopharyngeal carcinoma	Homo sapiens
GDS3622	Nrf2-deficient lung response to cigarette smoke: dose response and time course	Mus musculus
GDS3623	Heart regeneration in zebrafish	Danio rerio
GDS3639	Male and female fruit flies of various wild-type laboratory strains	Drosophila melanogaster
GDS3640	Copper effect on liver cell line: dose response and time course	Homo sapiens
GDS3644	Cerebral palsy: wrist muscles	Homo sapiens
GDS3646	Celiac disease: primary leukocytes	Homo sapiens
GDS3648	Cardiomyocyte response to various types of fatty acids in vitro	Rattus norvegicus
GDS3661	Hypertensive heart failure model	Rattus norvegicus
GDS3672	Hypertension model: aorta	Mus musculus
GDS3690	Atherosclerotic Coronary Artery Disease: circulating mononuclear cell types	Homo sapiens
GDS3715	Insulin effect on skeletal muscle	Homo sapiens
GDS3716	Breast cancer: histologically normal breast epithelium	Homo sapiens
GDS3703	Addictive drugs effect on brain striatum: time course	Mus musculus
GDS3707	Acute ethanol exposure: time course	Drosophila melanogaster
GDS3692	Lean B6.C-D7Mit353 strain: various tissues	Mus musculus

The 25 datasets obtained from the Gene Expression Omnibus (GEO) [25]. All datasets were retrieved between 4-04-2011 and 4-23-2011. Each dataset was log-transformed if it had not been already. For each dataset, four different correlation thresholds were used to build unweighted graphs.

From the expression data, we first constructed weighted graphs in which vertices represented probes and edge weights were Pearson correlation coefficients computed across experimental conditions. We then converted the weighted graphs into unweighted graphs by retaining only those edges whose weights were at or above some chosen threshold, *t*. For each dataset, we chose four values for *t*. All size/density values were within the spectrum typically seen in our work with biological datasets. The smallest graph had 3,828 vertices and 310,380 edges; the largest had 44,563 vertices and 2,052,228 edges.

**Figure 4:**
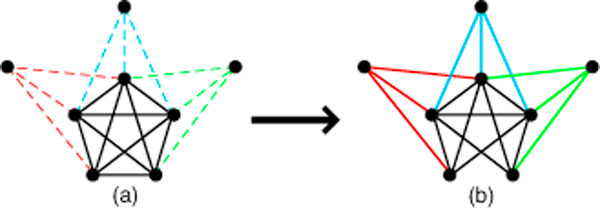
**Maximum Clique Sensitivity**. The number of maximum cliques in a graph can be highly subject to perturbations due, for example, to noise. For example, a graph may contain a single maximum clique *C *representing a putative network of size *k*, along with any number of vertices connected to *k *- 2 vertices in *C*. In (a), there is a single maximum clique of size *k *= 5, with "many" other vertices (only three are shown) connected to *k *- 2 = 3 of its nodes. In (b), noise results in the removal of a single edge, creating many maximum cliques now of size *k *- 1 = 4.

The number of maximum cliques for the graphs in our testbed ranged from 8 to 74486. As seen with our previous testbed, there was no discernible pattern based on graph size or density. One might ask why there is such wide, unpredictable variability. It turns out that the number of maximum cliques can be extremely sensitive to small changes in the graph. Even the modification of a single edge can have a huge effect. Consider, for example, a graph with a unique maximum clique of size *k*, along with a host of disjoint cliques of size *k *- 1. The removal of just one edge from what was the largest clique may now result in many maximum cliques of size *k *- 1. Edge addition can of course have similar effects. See Figure 4 for an illustrative example.

For each algorithm on each graph, we conducted timings on a dedicated node of a cluster to avoid interference from other processes. If the algorithm did not complete within 24 hours, it was halted and the graph was deemed to have not been solved. We chose thresholds to spread the runtimes of the graphs out over the five algorithms we were testing. The largest (smallest in the case of correlation p-value) threshold was selected so that a majority of the algorithms, if not all, solved the graph. The smallest (largest in the case of correlation p-value) threshold was selected so that at least one of the algorithms, but not all, solved the graph.

**Figure 5:**
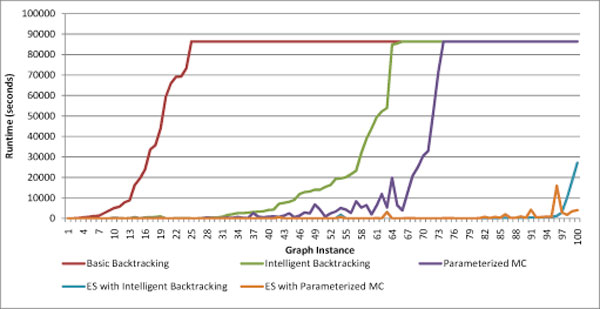
**Timings**. Timings on various approaches to MCE on the testbed of 100 biological graphs. Timings include all preprocessing, as well as the time to find the maximum clique size, where applicable. Runs were halted after 24 hours and deemed to have not been solved, as represented by those shown to take 86400 seconds. The graph instances are sorted first in order of runtimes for Basic Backtracking, then in order of runtimes for Intelligent Backtracking. This is a reasonable way to visualize the timings, though not perfect, since graphs that are difficult for one method may not be as difficult for another, hence the subsequent timings are not monotonic.

On each graph we timed the performance of Basic Backtracking, Intelligent Backtracking, and Paramaterized MC. We then reduced the graphs using ES and retested with Intelligent Backtracking and Parameterized MC, in which case the runtimes include both the reduction and the enumeration step. As expected, Basic Backtracking was found to be non-competitive. Both Intelligent Backtracking and Parameterized MC showed a distinct, often dramatic, improvement over Basic Backtracking. Figure 5 shows the runtimes of each of the five methods on all 100 test graphs. On some of the easier graphs, ones taking less than three minutes to solve, the overhead of ES actually caused a minor increase in the overall runtime. But on the more difficult instances its true benefit became apparent, reducing runtime by an order of magnitude or more. And in all cases where two or fewer algorithms solved the graph, the algorithm was either ES with Intelligent Backtracking, ES with Parameterized MC, or both.

## Conclusions

ES serves as a practical example of an innovative algorithm tailored to handle a difficult combinatorial problem by exploiting knowledge of the input space. It succeeds by exploiting properties of the graphs of interest, in this case the overlapping nature of maximum cliques. More broadly, these experiments underscore the importance of considering graph types when testing algorithms.

**Figure 6:**
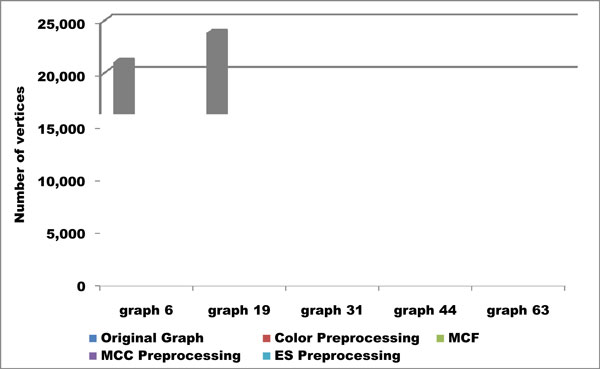
**Reduction in Graph Size**. Reduction in graph size thanks to preprocessing on five representative graphs chosen from our testbed. Each of the four preprocessing methods greatly reduces the graph size.

**Figure 7:**
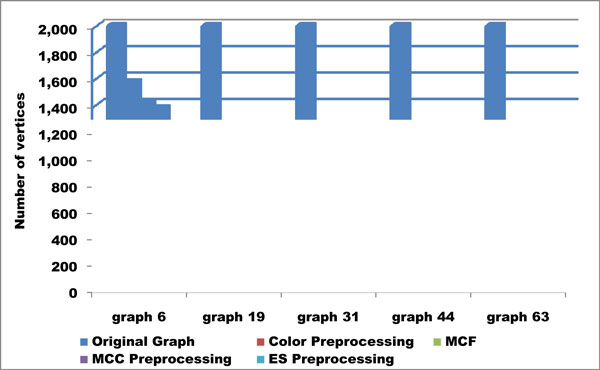
**Reduction in Graph Size**. A zoomed view of Figure 6, showing the effectiveness of each preprocessing method at reducing graph size. ES preprocessing results in the smallest reduced graph, often leaving only a small fraction of the vertices left by other methods.

It may be useful to examine graph size after applying MCC and ES, and compare to both the size of the original graph and the amount of reduction achieved by color preprocessing alone. Figures 6 and 7 depict original and reduced graph sizes for five graphs we originally tested.

While MCC seems as if it should produce better results, in practice we find it not to be the case for two reasons. First, the vertices in an MCC may collectively be connected to a large portion of the rest of the graph, and so very little reduction in graph size takes place. And second, any reduction in graph size may be redundant with FPT-style preprocessing rules already in place.

### Contrast to random graphs

It would have probably been fruitless to test and design our algorithms around random graphs. (Yet practitioners do just that with some regularity.) In fact it has long been observed that the topology of graphs derived from real relationships differs drastically from the Erdös-Rényi random graph model introduced in [26]. Attempts to characterize the properties of real data graphs have been made, such as the notion of scale-free graphs, in which the degrees of the vertices follow a power-law distribution [27]. While work to develop the scale-free model into a formal mathematical framework continues [28], there remains no generally accepted formal definition. More importantly, the scale-free model is an inadequate description of real data graphs. We have observed that constructing a graph so the vertices follow a power law (scale-free) degree distribution, but where edges are placed randomly otherwise using the vertex degrees as relative probabilities for edge placement, still results in graphs with numerous small disjoint maximum cliques. For instance, constructing graphs with the same degree distribution as each of the 75 biological graphs in our original testbed resulted in maximum clique sizes no greater than 5 for even the highest density graphs. Compare this to maximum clique sizes that ranged into hundreds of vertices in the corresponding biological graphs. Other metrics have been introduced to attempt to define important properties, such as cluster coefficient and diameter. Collectively, however, such metrics remain inadequate to model fully the types of graphs derived from actual biological data. The notions of maximum clique cover and essential vertices stem from the observation that transcriptomic data graphs tend to have one very large highly-connected region, and most (very often all) of the maximum cliques lie in that space. Furthermore, there tends to be a great amount of overlap between maximum cliques, perhaps as a natural result of gene pleiotropism. Such overlap is key to the runtime improvement achieved by the ES algorithm.

### Future research directions

Our efforts with MCE suggest a number of areas with potential for further investigation. A formal definition of the class of graphs for which ES achieves runtime improvements may lead to new theoretical complexity results, perhaps based upon parameterizing by the amount of maximum clique overlap. Furthermore, such a formal definition may form the basis of a new model for real data graphs. We have noted that the number of disjoint maximum cliques that can be extracted provides an upper bound on the size of an MCC. If we parameterize by the maximum clique size and the number of maximum cliques, does an FPT algorithm exist? In addition, formal mathematical results may be achieved on the sensitivity of the number of maximum cliques to small changes in the graph.

**Figure 8:**
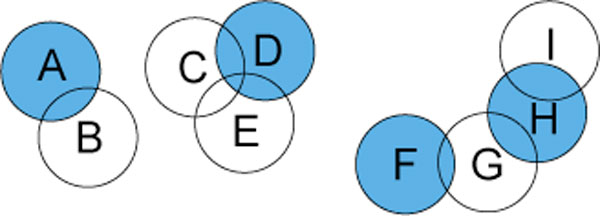
**The Subset Cover Problem**. The decision version of the subset cover problem asks if there are *k *or fewer subsets that cover all other subsets. A satisfying solution for *k *= 4 is the highlighted subsets.

Note that any MCC forms a hitting set over the set of maximum cliques, though not necessarily a minimum one. Also, a set *D *of disjoint maximum cliques, to which no additional disjoint maximum clique can be added, forms a *subset cover *over the set of all maximum cliques. That is, any maximum clique *C *∉ *D *contains at least one *v *∈ *D*. See Figure 8. To the best of our knowledge, this problem has not previously been studied. All we have found in the literature is one citation that erroneously reported it to be one of Karp's original NP-complete problems [29].

For the subset cover problem, we have noted that it is NP-hard by a simple reduction from hitting set. But in the context of MCE we have subsets all of the same size. It may be that this alters the complexity of the problem, or that one can achieve tighter complexity bounds when parameterizing by the subset size. Alternately, consider the problem of finding the minimum subset cover given a known minimum hitting set. The complexity of this tangential problem is not at all clear, although we conjecture it to be NP-complete in and of itself. Lastly, as a practical matter, exploring whether an algorithm that addresses the memory issues of the subset enumeration algorithm presented in [13] and improved in [14] may also prove fruitful. As we have found here, it may well depend at least in part on the data.

## Competing interests

The authors declare that they have no competing interests.

## Authors' contributions

JDE wrote the software employed in this study. CAP produced and collected problem instances from transcriptomic data and performed exhaustive timings. GLR provided background investigation and coordinated data presentation. MAL conceived of the project and coordinated manuscript preparation. All authors participated in algorithm design and analysis, and read and approved the final manuscript.
